# Psychometric assessment of proximal and distal concerns in the context of climate change

**DOI:** 10.1192/j.eurpsy.2025.2008

**Published:** 2025-08-26

**Authors:** A. T. Pereira, C. Cabaços, C. C. Marques, M. J. Soares, A. I. Araújo, A. Macedo

**Affiliations:** 1 Institute of Psychological Medicine, Faculty of Medicine University of Coimbra; 2 Institute of Psychologicam Medicine, Faculty of Medicine, University of Coimbra; 3 Coimbra Institute for Biomedical Imaging and Translational Research (CIBIT); 4 Institute for Biomedical Imaging and Translational Research (CIBIT), University of Coimbra, Coimbra; 5 Institute for Biomedical Imaging and Translational Research (CIBIT), University of Coimbra, Coimbraimbra, Portugal

## Abstract

**Introduction:**

Contemporary global crises, including Climate Change/CC, has increased the interest in Future Anxiety/FA. As an emotional response to the anticipation of threats in the distant rather than proximal future, FA is broader than worry, which generally focuses on particular issues and fluctuates depending on specific circumstances.

**Objectives:**

To analyze the psychometric properties of the Portuguese versions of Dark Future Scale (DFS; Zaleski et al. 2019), composed of 5 items to evaluate FA; and Climate Change Worry Scale (CCWS; Stewart 2021), a 10-items measure of proximal worry about CC; to explore whether they evaluate distinct or overlapping constructs.

**Methods:**

590 adults (64.6% women; mean age=34.40±16.18) answered DFS, CCWS (preliminary), Climate Change Distress and Impairment Scale/CC-DIS and Pro-Environmental Behaviours Scale/PEBS. Exploratory Factor Analysis (EFA; with a subsample of *n*=290) and Confirmatory Factor Analysis (CFA; *n*=300) were performed. DFS and CCWS structures tested with CFA were based on previous EFA results, including a model combining items from both scales.

**Results:**

**DFS:** CFA evidenced the good fit of the unidimensional model, χ2/df=3.314, CFI=.995, TLI=.990, GFI=.989, RMSEA=.060, *p*<.001; alfa=.91.

**CCWS:**

the unidimensional model (similar to the one found in the original version) (χ2/df=2.076; CFI=.982; TLI=.970; GFI=.965; RMSEA=.0590; *p*<.001; a=.90) and the two-factors model, with F1 composed of 6 items related to CC concerns and F2 of 4 items related to perceived interference (χ2/df=2.561; CFI=.973; TLI=.956; GFI=.956; RMSEA=.0611; p<.001; aF1=.75, aF2= .89) presented good fit.

**DFSandCCWS:**

the 2-factor model, with each scale being one factor (χ2/df=2.312; CFI=.962; TLI=.951; GFI=.928; RMSEA=.065; *p*<.001) and the 3-factor model, where CCWS divides into 2 factors (χ2/df=2.248; CFI=.964; TLI=.954; GFI=.926; RMSEA=.063; *p*<.001) resulted in good fit. FA correlated with CCWSTotal/F1/ F2 (*r*>.25). Correlations with CCDIS were *r*=.36 for FA and *r*<.55 for CCWSTotal/F1/F2; only CCWS total and dimensional scores correlated with PEB (*r*>.45) (all *p<.*001). When predicting CCDIS, FA adds 4% (R change, *p<.*001) to the variance explained by CCWS dimensions (R^2^=49.7%). Only CCWSConcerns (b=.345) but not CCWSInterference predicted PEB.

**Conclusions:**

DFS and CCWS Portuguese versions have adequate validity and reliability. Their moderate correlation and the validity of the measurement models tested suggest that they evaluate distinct constructs. FA seems more maladaptive than CCW: although it increments CC-DIS prediction, it does not correlate with PEBS. The same applies to CCWSInterference, which emphasizes that the CCWS two-factor structure may be useful to delimit CCW’s normal/pathological nature. We intend to use these scales in an ongoing research project on psychological factors associated with CC mitigation and adaptation.

**Disclosure of Interest:**

None Declared

